# Lifetime alcohol intake, drinking patterns over time and risk of stomach cancer: A pooled analysis of data from two prospective cohort studies

**DOI:** 10.1002/ijc.33504

**Published:** 2021-02-22

**Authors:** Harindra Jayasekara, Robert J. MacInnis, Leila Lujan‐Barroso, Ana‐Lucia Mayen‐Chacon, Amanda J. Cross, Bengt Wallner, Domenico Palli, Fulvio Ricceri, Valeria Pala, Salvatore Panico, Rosario Tumino, Tilman Kühn, Rudolf Kaaks, Kostas Tsilidis, Maria‐Jose Sánchez, Pilar Amiano, Eva Ardanaz, María Dolores Chirlaque López, Susana Merino, Joseph A. Rothwell, Marie‐Christine Boutron‐Ruault, Gianluca Severi, Hanna Sternby, Emily Sonestedt, Bas Bueno‐de‐Mesquita, Heiner Boeing, Ruth Travis, Torkjel M. Sandanger, Antonia Trichopoulou, Anna Karakatsani, Eleni Peppa, Anne Tjønneland, Yi Yang, Allison M. Hodge, Hazel Mitchell, Andrew Haydon, Robin Room, John L. Hopper, Elisabete Weiderpass, Marc J. Gunter, Elio Riboli, Graham G. Giles, Roger L. Milne, Antonio Agudo, Dallas R. English, Pietro Ferrari

**Affiliations:** ^1^ Cancer Epidemiology Division Cancer Council Victoria Melbourne Victoria Australia; ^2^ Centre for Epidemiology and Biostatistics Melbourne School of Population and Global Health, The University of Melbourne Melbourne Victoria Australia; ^3^ Centre for Alcohol Policy Research La Trobe University Bundoora Victoria Australia; ^4^ Unit of Nutrition and Cancer, Catalan Institute of Oncology ‐ ICO, Nutrition and Cancer Group, Bellvitge Biomedical Research Institute ‐ IDIBELL, L'Hospitalet de Llobregat Barcelona Spain; ^5^ Department of Nursing of Public Health Mental Health and Maternity and Child Health School of Nursing Universitat de Barcelona Barcelona Spain; ^6^ Nutritional Methodology and Biostatistics Group, International Agency for Research on Cancer, World Health Organization Lyon France; ^7^ Department of Epidemiology and Biostatistics School of Public Health, Imperial College London London UK; ^8^ Department of Surgical and Perioperatve Sciences, Surgery Umeå University Hospital Umeå Sweden; ^9^ Cancer Risk Factors and Life‐Style Epidemiology Unit Institute for Cancer Research, Prevention and Clinical Network – ISPRO Florence Italy; ^10^ Department of Clinical and Biological Sciences University of Turin Turin Italy; ^11^ Unit of Epidemiology, Regional Health Service ASL TO3 Grugliasco Italy; ^12^ Epidemiology and Prevention Unit, Fondazione IRCCS Istituto Nazionale dei Tumori di Milano Milan Italy; ^13^ Dipartimento di Medicina Clinica e Chirurgia Federico II University Naples Italy; ^14^ Cancer Registry and Histopathology Department Provincial Health Authority (ASP) Ragusa Italy; ^15^ Division of Cancer Epidemiology German Cancer Research Center (DKFZ) Heidelberg Germany; ^16^ Escuela Andaluza de Salud Pública (EASP) Granada Spain; ^17^ Instituto de Investigación Biosanitaria ibs.GRANADA Granada Spain; ^18^ CIBER in Epidemiology and Public Health (CIBERESP) Madrid Spain; ^19^ Universidad de Granada Granada Spain; ^20^ Public Health Division of Gipuzkoa, BioDonostia Research Institute Donostia‐San Sebastian Spain; ^21^ Navarra Public Health Institute Pamplona Spain; ^22^ IdiSNA, Navarra Institute for Health Research Pamplona Spain; ^23^ Department of Epidemiology Regional Health Council, IMIB‐Arrixaca, Murcia University Murcia Spain; ^24^ Public Health Directorate, Regional Government of Asturias Oviedo Spain; ^25^ CESP (U1018), Faculté de médecine Université Paris‐Saclay, UVSQ, INSERM Villejuif France; ^26^ Gustave Roussy Villejuif France; ^27^ Department of Statistics Computer Science and Applications “G. Parenti” (DISIA), University of Florence Florence Italy; ^28^ Department of Surgery Institution of Clinical Sciences Malmö, Lund University Lund Sweden; ^29^ Nutritional Epidemiology, Department of Clinical Sciences Malmö Lund University Malmö Sweden; ^30^ Department for Determinants of Chronic Diseases (DCD), National Institute for Public Health and the Environment (RIVM) Bilthoven The Netherlands; ^31^ Institute of Nutrition Science, University of Potsdam Nuthetal Germany; ^32^ Cancer Epidemiology Unit, Nuffield Department of Population Health University of Oxford Oxford UK; ^33^ Department of Community Medicine, Faculty of Health Sciences UiT‐the Arctic University of Norway Tromsø Norway; ^34^ Hellenic Health Foundation Athens Greece; ^35^ 2nd Pulmonary Medicine Department School of Medicine, National Kapodistrian University of Athens, “ATTIKON” University Hospital Haidari Greece; ^36^ Danish Cancer Society Research Center Copenhagen Denmark; ^37^ Department of Public Health University of Copenhagen Copenhagen Denmark; ^38^ School of Biotechnology and Biomolecular Sciences, University of New South Wales Kensington New South Wales Australia; ^39^ Department of Medical Oncology Alfred Hospital Melbourne Victoria Australia; ^40^ Centre for Social Research on Alcohol and Drugs, Department of Public Health Sciences Stockholm University Stockholm Sweden; ^41^ Office of the Director, International Agency for Research on Cancer, World Health Organization Lyon France; ^42^ Nutritional Epidemiology Group, International Agency for Research on Cancer, World Health Organization Lyon France; ^43^ Precision Medicine, School of Clinical Sciences at Monash Health, Monash University Clayton Victoria Australia

**Keywords:** cardia cancer, EPIC, lifetime alcohol intake, MCCS, noncardia cancer, stomach cancer

## Abstract

Alcohol consumption is causally linked to several cancers but the evidence for stomach cancer is inconclusive. In our study, the association between long‐term alcohol intake and risk of stomach cancer and its subtypes was evaluated. We performed a pooled analysis of data collected at baseline from 491 714 participants in the European Prospective Investigation into Cancer and Nutrition and the Melbourne Collaborative Cohort Study. Hazard ratios (HRs) and 95% confidence intervals (CIs) were estimated for incident stomach cancer in relation to lifetime alcohol intake and group‐based life course intake trajectories, adjusted for potential confounders including *Helicobacter pylori* infection. In all, 1225 incident stomach cancers (78% noncardia) were diagnosed over 7 094 637 person‐years; 984 in 382 957 study participants with lifetime alcohol intake data (5 455 507 person‐years). Although lifetime alcohol intake was not associated with overall stomach cancer risk, we observed a weak positive association with noncardia cancer (HR = 1.03, 95% CI: 1.00‐1.06 per 10 g/d increment), with a HR of 1.50 (95% CI: 1.08‐2.09) for ≥60 g/d compared to 0.1 to 4.9 g/d. A weak inverse association with cardia cancer (HR = 0.93, 95% CI: 0.87‐1.00) was also observed. HRs of 1.48 (95% CI: 1.10‐1.99) for noncardia and 0.51 (95% CI: 0.26‐1.03) for cardia cancer were observed for a life course trajectory characterized by *heavy decreasing intake* compared to *light stable intake* (*P*_homogeneity_ = .02). These associations did not differ appreciably by smoking or *H pylori* infection status. Limiting alcohol use during lifetime, particularly avoiding heavy use during early adulthood, might help prevent noncardia stomach cancer. Heterogeneous associations observed for cardia and noncardia cancers may indicate etiologic differences.

## Abstract 2

What's new?

Alcohol consumption has been causally linked to several cancers, but results for stomach cancer have been inconclusive. In this large, international study, the authors found a positive association between long‐term, heavy drinking and non‐cardia stomach cancer. This association persisted when data were adjusted for potentially confounding factors such as smoking, body mass index, and *Helicobacter pylori* infection. A weak inverse association was observed with cardia cancer, suggesting that there may be etiologic differences between non‐cardia vs. cardia subtypes.

**Abbreviations**
CI: confidence interval
EPIC: European Prospective Investigation into Cancer and Nutrition
HR: hazard ratio
ICD‐O‐3: International Classification of Diseases for Oncology
MCCS: Melbourne Collaborative Cohort Study
VCR: Victorian Cancer Registry

## 1 INTRODUCTION

Stomach cancer is the fifth most common cancer with an estimated 1 033 701 incident cases (5.7% of all cancers) worldwide in 2018.^1^ Due to its high fatality, it is the third leading cause of death from cancer,^1^ with modest survival even in high‐income countries.^2^ The absence of specific symptoms or a marker for early detection often leads to diagnosis when the tumor is already locally advanced or metastatic. Most stomach cancers are potentially preventable: for example, 77% of stomach cancer deaths and 65% of cases in Australia in 2013 were estimated to be attributable to modifiable risk factors.^3^ The vast majority of stomach cancers are known to be associated with infectious agents such as the bacterium *Helicobacter pylori* and Epstein‐Barr virus^4^; cigarette smoking and industrial chemical exposure are established risk factors while other modifiable lifestyle factors, including alcohol use, consumption of processed meat, foods preserved by salting, and obesity, are classified as *probable* causes of stomach cancer.^5^

Alcohol drinking caused an estimated 3 million deaths globally (5.3% of all deaths) and an estimated 400 000 deaths from cancer (representing 4.2% of all cancer deaths) in 2016.^6^ Ethanol in alcoholic beverages and its metabolites are causally linked to cancers of the oral cavity, pharynx, larynx, esophagus (squamous‐cell carcinoma), liver, colorectum and female breast.^7^ A role for alcohol use in the etiology of stomach cancer is plausible but the epidemiological evidence remains equivocal.^5^, ^8^ Most evidence from prospective studies is based on consumption data that refer to the time of study recruitment, that is, alcohol intake at baseline. The baseline intake might not be representative of participants' consumption during earlier age periods, particularly for heavy drinkers who had reduced alcohol consumption.

In our study, we estimated associations for baseline and lifetime alcohol use with risk of stomach cancer and its subtypes, using retrospective information on consumption at various ages before recruitment in two large prospective studies.

## 2 MATERIALS AND METHODS

A pooled analysis of 491 714 participants in the European Prospective Investigation into Cancer and Nutrition (EPIC) and the Melbourne Collaborative Cohort Study (MCCS) was conducted.

### 2.1 European prospective investigation into cancer and nutrition

#### 2.1.1 Participants

**FIGURE 1:**
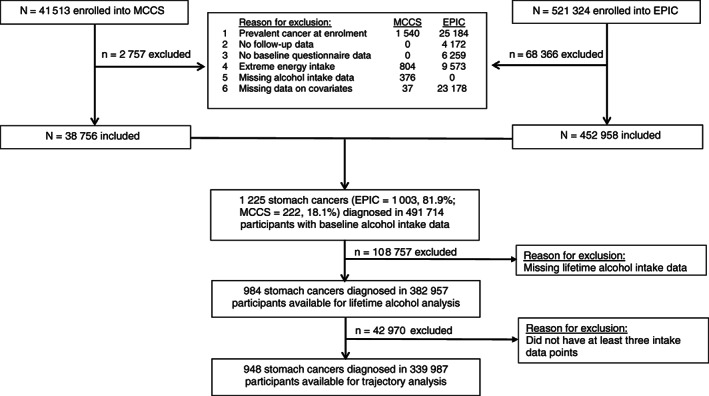
Flow diagram showing selection of participants. EPIC, European Prospective Investigation into Cancer and Nutrition; MCCS, Melbourne Collaborative Cohort Study

EPIC is a multicenter prospective cohort study of 521 324 participants (70.6% women), mostly of Caucasian descent, aged 35 to 70 years when enrolled, predominantly during 1992to 1998, designed to investigate the relationship among dietary habits, nutritional status and various lifestyle/environmental factors and cancer incidence.^9^ EPIC has 23 centers in 10 European countries (Denmark, France, Greece, Germany, Italy, the Netherlands, Norway, Spain, Sweden and the United Kingdom). Eligible participants gave written informed consent. Ethical review boards from the International Agency for Research on Cancer and local centers participating in EPIC approved the study. Participants were excluded if they had a diagnosis of cancer before recruitment (n = 25 184), had no follow‐up information (n = 4172), no baseline questionnaire data (n = 6259), had extreme energy intake (n = 9573) or were missing data on the covariates modeled (n = 23 178). Of the remaining 452 958 (86.9% of the total cohort) participants, 108 461 (24.0%) had no information on lifetime alcohol intake (Figure 1).

#### 2.1.2 Data collection

Diet over the 12 months before enrolment was measured by validated country‐specific dietary questionnaires^9^ designed to capture local dietary habits.^10^ Most centers adopted a self‐administered dietary questionnaire with 88 to 266 food items. Baseline height and weight (self‐reported in France, Norway and the UK Oxford center, measured elsewhere), alcohol intake, smoking status, occupational physical activity and medical history were collected with questionnaires. *H pylori* status was determined in nested case‐control studies on stomach cancer^11^ by using a combination of ELISA (Pyloriset EIA‐GIII kit; Orion Diagnostics) and immunoblotting^12^ assays in plasma collected at baseline. Cases and controls were matched on age group, sex, center and date of blood collection. Details on establishing *H pylori*‐seropositivity have been published previously.^11^

#### 2.1.3 Assessment of alcohol intake

Alcohol intake at baseline was estimated from validated dietary questionnaires where participants reported the number of standard glasses of wine, beer, cider, sweet liquor, distilled spirits and fortified wines they consumed daily or weekly during the 12 months before recruitment.^9^, ^10^ For each participant, an average daily alcohol intake expressed in grams per day was calculated based on the standard glass volume and ethanol content for each type of alcoholic beverage for each country using information collected through 24‐hour dietary recalls from a subgroup of the cohort.^13^, ^14^ Study participants also reported their alcohol consumption at 20, 30, 40 and 50 years of age (as appropriate for age at baseline).^15^ The average lifetime alcohol intake was calculated as a weighted average of intakes at different ages with weights equal to the time of exposure to alcohol at different ages.^16^ Information on adult lifetime alcohol intake was available for approximately 76% of participants.^16^

#### 2.1.4 Cohort follow‐up and ascertainment of cases and deaths

Cases were identified from population cancer registries except in France, Germany, Greece and Naples (Italy), where a combination of different methods, including accessing health insurance records, hospital‐based cancer and pathology registries and active follow‐up (participant tracking), were used. Incident primary invasive stomach cancer cases were defined as those with code C16 according to the 10th revision of the International Classification of Diseases (C16.0, cardia; C16.1, fundus of stomach; C16.2, body of stomach; C16.3, pyloric antrum; C16.4, pylorus; C16.5, lesser curvature of stomach, not classifiable to C16.1‐C16.4; C16.6, greater curvature of stomach, not classifiable to C16.0‐C16.4; C16.8, overlapping lesion of stomach; C16.9, stomach unspecified). Histologic type^17^ (diffuse‐type [morphology codes 8145/3, 8490/3, 8142/3], intestinal‐type [morphology codes 8144/3, 8211/3, 8260/3, 8480/3, 8481/3, 8140/3]; other morphology codes classified as mixed/other/unknown)^18^, ^19^ and anatomical location (cardia C16.0, noncardia C16.1‐6, overlapping/unspecified C16.8‐9) were determined; a panel of pathologists reviewed original pathology reports, tumor slides and paraffin blocks for a subset of cases.^20^

### 2.2 Melbourne Collaborative Cohort Study

#### 2.2.1 Participants

The MCCS is a prospective cohort study of 41 513 people (58.9% women; 99.2% aged 40‐69 years), all of white European descent, recruited during 1990to 1994 in Melbourne, Australia.^21^ The study protocol was approved by the Cancer Council Victoria Human Research Ethics Committee. Participants gave written informed consent to participate and for investigators to obtain access to their medical records. For this analysis, we selected participants who did not have a previous diagnosis of cancer at enrolment (n = 39 973). Participants reporting extreme values of total energy intake (<1st percentile and >99th percentile) (n = 804) or missing alcohol consumption data (n = 376), or missing data on any of the covariates modeled (n = 37) were excluded, leaving 38 756 (93.4% of the total cohort) eligible for this analysis; only 296 (0.8) were missing information on lifetime alcohol intake (Figure 1).

#### 2.2.2 Baseline data collection

Structured interview schedules were used to obtain information on potential risk factors including age, sex, country of birth, education, previous medical conditions and lifestyle behaviors (including cigarette smoking, physical activity and alcohol intake). A 121‐item food frequency questionnaire was used to collect dietary information.^22^ Height was measured to 1 mm with a stadiometer, and weight to 100 g using digital electronic scales. Residential address was used to classify participants into quintiles of an area‐based measure of socioeconomic status. *H pylori* status was determined in nested case‐control studies of stomach cancer^23^, ^24^ by using immunoblotting^12^ assays in plasma collected at baseline. Cases and controls were matched on year of birth, sex and country of birth. Details on establishing *H pylori*‐seropositivity have been published previously for MCCS^24^ participants.

#### 2.2.3 Assessment of alcohol intake

Participants were asked at baseline if they had ever drunk at least 12 alcoholic drinks in a year. Those who had (“nonlifetime abstainers”) were then asked about their usual frequency of consumption and usual quantity consumed per drinking occasion for beer, wine and spirits separately during 10‐year age periods commencing at age 20, up to the decade of their age at baseline attendance. Usual intake within each age period in grams per day for each beverage type was calculated by multiplying intake frequency by quantity and standard amount of alcohol per container using Australian food composition tables.^25^ The alcohol intake for each age period in grams per day was calculated as the sum of intake from the three beverage types. The baseline alcohol intake in grams per day was obtained from intake for the age period encompassing baseline. Beverage‐specific total intakes within age periods were summed to obtain total lifetime intakes in grams. The average lifetime alcohol intake in grams per day was derived by dividing the total lifetime intake by the total number of days within the age intervals up to baseline attendance.

#### 2.2.4 Cohort follow‐up and ascertainment of cases and deaths

Cases and vital status were ascertained through the Victorian Cancer Registry (VCR), the Victorian Registry of Births, Deaths and Marriages, the National Death Index and the Australian Cancer Database. The outcome was defined as a histopathological diagnosis of primary invasive adenocarcinoma of the stomach, coded following the 3rd Revision of the International Classification of Diseases for Oncology (ICD‐O‐3) as C16. Tumor histopathology and anatomical site were obtained from the VCR.

### 2.3 Statistical analysis

The association between alcohol intake and stomach cancer was examined after pooling EPIC and MCCS data. Cox regression^26^ was used to estimate hazard ratios (HRs) and 95% confidence intervals (CIs) with age as the time scale and stratified by year of birth (<1925, 5‐year categories from 1925 to 1964, ≥1965) and study center (EPIC) or country of birth (MCCS, Australia/New Zealand/United Kingdom or Italy/Greece). The age when the baseline questionnaire was returned (EPIC) or of baseline attendance (MCCS) was defined as the age when follow‐up started. Follow‐up was censored at diagnosis of any first primary cancer, death, emigration or end of follow‐up (center‐specific censor date for EPIC; 31 January 2017 for MCCS).

Alcohol intake was modeled as a continuous variable (per 10 g/d increment in intake, the size of a standard drink in Australia) and for intake categories derived using the following cut‐points: nondrinkers, 0.1‐4.9, 5‐14.9, 15‐29.9, 30‐59.9, ≥60 g/d for total alcohol; nondrinkers, 0.1‐4.9, 5‐14.9, 15‐29.9, ≥30 g/d for beer and wine; and nondrinkers, 0.1‐4.9, 5‐14.9, ≥15 g/d for spirits. Consistently throughout our study, the 0.1‐4.9 g/d category (ie, very light or occasional drinkers) was used as the reference group. Nearly 78% of the eligible study participants (n = 382 957) were included in analyses for lifetime alcohol intake (Figure 1). Based on this information, 35% of nondrinkers at baseline were former drinkers. A causal diagram (directed acyclic graph) and existing evidence^5^ guided the inclusion of confounding variables in multivariable models: sex, education (primary school, technical school, secondary school, university), cigarette smoking (never, former >10 years since quitting, former ≤10 years since quitting; current <20 cigarettes/d, current ≥20 cigarettes/d, other), body mass index (kg/m^2^), intake of total red and processed meat (g/d), fruit intake (g/d) and total energy from food not including alcoholic beverages (Kcal/d) (Supplementary Figure 1). The evaluation of *H pylori* infection status as a confounder for noncardia cancer is described below.

We also examined associations with patterns of lifetime alcohol intake based on a semiparametric group‐based trajectory model.^27^, ^28^ This model is an application of finite mixture modeling which assumes the study sample is composed of a mixture of groups following homogenous courses.^27^ Longitudinal alcohol intake data were fitted as a mixture of several latent trajectories in a censored normal model, allowing for the lower (zero g/d) and upper intake (capped at 100 g/d) limits, with a polynomial function for age.^28^ We used the Bayesian information criterion and the log Bayes factor to select optimal shapes and number of trajectory groups through a two‐stage approach.^27^ First, the number of groups was determined assuming all trajectory groups were cubic functions of age. Second, the preferred order of the polynomial (ie, quadratic or cubic) for each trajectory was determined. Participants were assigned to the group for which their posterior predicted probability calculated from the final model was largest. Model adequacy was evaluated using recommended diagnostic measures: average posterior probability of assignment for each group of 0.7 or higher; odds of correct classification of 5.0 or higher; the proportion of a sample assigned to a certain group close to the proportion estimated from the model; and a reasonably narrow CI around each trajectory.^27^ Participants with at least three intake data points (n = 339, 987; 69% of the eligible study participants) were included (Figure 1). We repeated this analysis excluding former drinkers at baseline.

To test for heterogeneity in the HRs between anatomical subsites (cardia and noncardia) and histologic subtypes (diffuse‐type and intestinal‐type) of stomach cancer, Cox regression models were fit in competing risks analysis.^29^ An augmented data set was created where the initial data set was replicated a number of times equal to the different subtypes. In each replicated data set, the competing subtype was set to censored observations and the analyses were stratified by the endpoint type.^30^ A histopathologic validation of a subset of EPIC data (n = 373 cases) found that all cancers classified as “mixed site,” and over half of the cancers classified as “gastric unknown,” were in fact noncardia cancers.^20^ Because most tumors with site codes C16.8 (overlapping) and C16.9 (unspecified) are noncardia cancers, we classified tumors with site codes C16.1‐6, C16.8 and C16.9 as noncardia as has been done previously.^31^, ^32^

A dose‐response relationship between lifetime alcohol intake and stomach cancer incidence was examined by comparing models that included alcohol as a linear term and with restricted cubic splines (with five knots placed at 0.1, 5, 30, 60 and 100 g/d), with upper intake capped at 100 g/d.^33^ We evaluated potential effect modification by sex, cigarette smoking and body mass index by including interaction terms with lifetime alcohol intake (continuous). We assessed the interaction term with the likelihood ratio test. Interaction with *H pylori* infection status and confounding due to it were evaluated for noncardia cancer using pooled data from EPIC^11^ and MCCS^23^, ^24^ nested case‐control studies (374 noncardia cancer cases and 1163 controls). Conditional logistic regression models included education, cigarette smoking, body mass index, meat intake, fruit intake and energy intake from food.

Sensitivity analyses were performed (a) excluding the first 2 years of follow‐up, (b) without adjustment for body mass index and dietary covariates and (c) by estimating study‐specific HRs and pooling study‐specific results using random effects models. Each model was examined for outliers and influential points. Nested models were compared using likelihood ratio tests. Tests based on Schoenfeld residuals showed no departure from the proportional hazard assumptions. All statistical tests were two‐sided, and statistical analyses were performed using Stata 16.1 (StataCorp, College Station, TX).

## 3 RESULTS

Characteristics of the 491 714 participants are described in Table 1. EPIC contributed 92.1% of participants with a mean follow‐up of 14 years and MCCS had a mean follow‐up of 20 years, with 1225 incident stomach cancer cases from both studies combined (cardia C16.0 = 274, 22.4%; noncardia = 951, 77.6% including C16.1‐16.6 = 484, 39.5% and C16.8‐16.9 = 467, 38.1%). Over two‐thirds of participants were women, but more cases (n = 690, 56.3%) were men. The mean age at recruitment was 51.4 years; cases were diagnosed at a mean age of 67 years. Stomach cancer incidenced by country for EPIC and by country of birth for MCCS are reported in Supplementary Table 1. EPIC data for France and Norway only included women hence their lower incidence.

**TABLE 1:** Characteristics of study participants from the European Prospective Investigation into Cancer and Nutrition (EPIC) and the Melbourne Collaborative Cohort Study (MCCS)

	EPIC	MCCS	Total
Age at recruitment (years), mean (range)^a^	51.1 (33.3‐65.7)	55.2 (41.5‐68.3)	51.4 (34.2‐66.3)
Sex, n (%)			
Male	137 138 (30.3)	15 804 (40.8)	152 942 (31.1)
Female	315 820 (69.7)	22 952 (59.2)	338 772 (68.9)
Education, n (%)			
Primary school	141 125 (31.2)	7461 (19.3)	148 586 (30.2)
Technical school	104 761 (23.1)	14 746 (38.0)	119 507 (24.3)
Secondary school	95 300 (21.0)	8014 (20.7)	103 314 (21.0)
University	111 772 (24.7)	8535 (22.0)	120 307 (24.5)
Cigarette smoking intensity, n (%)			
Never	197 812 (43.7)	22 447 (57.9)	220 259 (44.8)
Former >10 years since quitting	57 609 (12.7)	7618 (19.7)	80 371 (16.3)
Former ≤10 years since quitting	34 106 (7.5)	4312 (11.1)	48 518 (9.9)
Current <20 cigarettes/d	44 206 (9.8)	1986 (5.1)	59 595 (12.1)
Current ≥20 cigarettes/d	72 753 (16.1)	2285 (5.9)	36 391 (7.4)
Other (incomplete, pipes, other)	46 472 (10.2)	108 (0.3)	46 580 (9.5)
Baseline alcohol intake (g/d), n (%)			
Abstainer	61 759 (13.6)	15 237 (39.3)	76 996 (15.7)
0.1‐4.9	155 372 (34.3)	7290 (18.8)	162 662 (33.1)
5‐14.9	120 777 (26.7)	5684 (14.7)	126 461 (25.7)
15‐29.9	62 751 (13.9)	5488 (14.2)	68 239 (13.9)
30‐59.9	40 957 (9.0)	3883 (10.0)	44 840 (9.1)
≥60	11 342 (2.5)	1174 (3.0)	12 516 (2.5)
Lifetime alcohol intake (g/d), n (%)			
Lifetime abstainer	27 370 (6.0)	11 082 (28.6)	38 452 (7.8)
0.1‐4.9	120 585 (26.6)	8184 (21.1)	128 769 (26.2)
5‐14.9	104 415 (23.0)	8493 (21.9)	112 908 (23.0)
15‐29.9	55 552 (12.3)	6065 (15.6)	61 617 (12.5)
30‐59.9	27 023 (6.0)	3625 (9.4)	30 648 (6.2)
≥60	9552 (2.1)	1011 (2.6)	10 563 (2.2)
Missing	108 461 (24.0)	296 (0.8)	108 757 (22.1)
Body mass index (kg/m^2^), mean (range)^a^	25.4 (19.7‐33.2)	26.9 (20.8‐35.0)	25.6 (19.8‐33.4)
Total red and processed meat intake (g/d), mean (range)^a^	75.6 (3.4‐168.1)	123.7 (32.5‐260.2)	79.4 (4.4‐177.7)
Fruit intake (g/d), mean (range)^a^	238.9 (32.5‐571.7)	450.0 (81.8‐1041.6)	255.5 (34.3‐626.0)
Energy intake from food (Kcal/d), mean (range)^a^	1994 (1158‐3082)	2097 (1117‐3501)	2002 (1155‐3112)
Total participants, n	452 958	38 756	491 714

^a^ Range = 5th to 95th percentile.

About one‐third of participants was either lifetime abstainers or drank less than 5 g/d, and 2.2% reported a lifetime intake of ≥60 g/d (Table 1). Although the proportion of lifetime abstainers was higher for MCCS (28.6%) than EPIC (6%), the median lifetime alcohol intake for drinkers was higher for the MCCS than EPIC (Supplementary Table 2). Male drinkers consumed more alcohol during their lifetime than female drinkers (median intakes 18.4 and 5.1 g/d, respectively) (Supplementary Table 2).

### 3.1 Lifetime alcohol intake and stomach cancer incidence

Lifetime alcohol intake was not associated with overall stomach cancer incidence (HR = 1.01, 95% CI: 0.99‐1.04 for a 10 g/d increment) (Table 2). The HR for a lifetime intake of 60 g/d or greater was 1.19 (95% CI: 0.88‐1.61), compared to 0.1 to 4.9 g/d. Models with cubic splines did not fit better than models with a single linear term for lifetime intake (*P* = .94). In analyses for subsites of the stomach, a 10 g/d increment in lifetime alcohol intake was weakly associated with increased incidence of noncardia cancer (HR = 1.03, 95% CI: 1.00‐1.06; *P* = .03) and showed a weak inverse association with cancer of the gastric cardia (HR = 0.93, 95% CI: 0.87‐1.00 for a 10 g/d increment; *P* = .06) (*P*_homogeneity_ < .01) (Table 2; Supplementary Figure 2). Using intake categories, for a lifetime intake of 60 g/d or greater, the HR was 1.50 (95% CI: 1.08‐2.09), compared to 0.1‐4.9 g/d, for noncardia cancer; the corresponding HR for cardia cancer was 0.48 (95% CI: 0.23‐1.01) (*P*_homogeneity_ = .03) (Table 2). When stratified by sex, HRs were similar for men and women for overall and noncardia stomach cancer (*P*_interaction_ = .71 and *P*_interaction_ = .60, respectively), and the inverse association with cardia cancer was observed in men only (*P*_interaction_ = .02) (Table 3; Supplementary Table 3).

**TABLE 2:** Hazard ratios for overall and site‐specific stomach cancer for baseline and lifetime alcohol intake in the European Prospective Investigation into Cancer and Nutrition (EPIC) and the Melbourne Collaborative Cohort Study (MCCS)

	All stomach cancer				By subsite						
					Cancer of the gastric cardia^a^		Noncardia cancer^a^				
	Person‐years	Cases n (%)	HR (95% CI)^c^	*P*_trend_ ^d^	Cases n (%)	HR (95% CI)^e^	*P*_trend_ ^d^	Cases n (%)	HR (95% CI)^e^	*P*_trend_ ^d^	*P*_homogeneity_ ^b^
Baseline alcohol intake											
For a 10 g/d increment	7 094 637	1225 (100)	1.01 (0.98‐1.04)	.62	274 (22.4)	1.01 (0.96‐1.07)	.63	951 (77.6)	1.01 (0.97‐1.04)	.75	.80
Intake categories				.88							.75
Abstainers	1 173 928	234 (19.1)	1.01 (0.85‐1.22)		36 (13.1)	0.81 (0.54‐1.23)		198 (20.8)	1.06 (0.87‐1.29)		
0.1‐4.9	2 322 506	321 (26.2)	1.00		69 (25.2)	1.00		252 (26.5)	1.00		
5‐14.9	1 814 195	252 (20.6)	0.92 (0.77‐1.09)		59 (21.5)	0.91 (0.64‐1.29)		193 (20.3)	0.92 (0.76‐1.11)		
15‐29.9	972 048	194 (15.8)	1.01 (0.83‐1.22)		50 (18.3)	1.03 (0.71‐1.50)		144 (15.1)	0.99 (0.80‐1.23)		
30‐59.9	639 349	163 (13.3)	1.05 (0.85‐1.29)		46 (16.8)	1.12 (0.75‐1.65)		117 (12.3)	1.02 (0.80‐1.29)		
≥60	172 611	61 (5.0)	1.06 (0.79‐1.43)		14 (5.1)	0.91 (0.50‐1.65)		47 (5.0)	1.12 (0.80‐1.56)		
Lifetime alcohol intake											
For a 10 g/d increment	5 455 507	984 (100)	1.01 (0.99‐1.04)	.37	224 (22.8)	0.93 (0.87‐1.00)	.06	760 (77.2)	1.03 (1.00‐1.06)	.03	<.01
Intake categories				.66							.03
Abstainers	604 408	114 (11.6)	0.91 (0.72‐1.16)		15 (6.7)	0.58 (0.33‐1.04)		99 (13.0)	1.00 (0.77‐1.29)		
0.1‐4.9	1 794 961	244 (24.8)	1.00		58 (25.9)	1.00		186 (24.5)	1.00		
5‐14.9	1 600 337	224 (22.8)	0.84 (0.69‐1.01)		57 (25.5)	0.76 (0.52‐1.10)		167 (22.0)	0.86 (0.69‐1.07)		
15‐29.9	878 795	207 (21.0)	1.08 (0.88‐1.33)		58 (25.9)	0.98 (0.66‐1.46)		149 (19.6)	1.11 (0.87‐1.40)		
30‐59.9	433 250	123 (12.5)	0.90 (0.70‐1.15)		27 (12.0)	0.62 (0.38‐1.02)		96 (12.6)	1.02 (0.77‐1.35)		
≥60	143 756	72 (7.3)	1.19 (0.88‐1.61)		9 (4.0)	0.48 (0.23‐1.01)		63 (8.3)	1.50 (1.08‐2.09)		

Abbreviations: CI, confidence interval; HR, hazard ratio.

^a^ Cardia (C16.0) and noncardia (C16.1‐6, C16.8, C16.9).

^b^ Test of homogeneity using the likelihood ratio test.

^c^ Adjusted for age, sex, education (primary school, technical school, secondary school, university), cigarette smoking (never, former >10 years since quitting, former ≤10 years since quitting; current <20 cigarettes/d, current ≥20 cigarettes/d, other), body mass index (kg/m^2^), total red and processed meat intake (g/d), fruit intake (g/d) and total energy from food not including alcoholic beverages (Kcal/d), and stratified by birth cohort (year of birth <1925, 5‐year categories for 1925 to 1964, ≥1965) and center (center in EPIC, two categories for individuals born in Australia/New Zealand/United Kingdom or Italy/Greece in MCCS).

^d^ Wald test from Cox regression models assessing linear trends for a 10 g/d increment in alcohol intake and for intake categories as a continuous measure.

^e^ Adjusted for age, sex, education (primary school, technical school, secondary school, university), cigarette smoking (never, former >10 years since quitting, former ≤10 years since quitting; current <20 cigarettes/d, current ≥20 cigarettes/d, other), body mass index (kg/m^2^), total red and processed meat intake (g/d), fruit intake (g/d) and total energy from food not including alcoholic beverages (Kcal/d), and stratified by birth cohort (year of birth <1925, 5‐year categories for 1925 to 1964, ≥1965) and center (center in EPIC, two categories for individuals born in Australia/New Zealand/United Kingdom or Italy/Greece in MCCS); interaction terms were also fit for sex, cigarette smoking and education in the models.

**TABLE 3:** Hazard ratios for overall and site‐specific stomach cancer for a 10 g/d increment in lifetime alcohol intake by sex, smoking status and body mass index (BMI) in the European Prospective Investigation into Cancer and Nutrition (EPIC) and the Melbourne Collaborative Cohort Study (MCCS)

	All stomach cancer					By subsite							
						Cancer of the gastric cardia^a^				Noncardia cancer^a^			
	Person‐years	Cases n (%)	HR (95% CI)^b^	*P*_trend_ ^c^	*P*_interaction_	Cases n (%)	HR (95% CI)^d^	*P*_trend_ ^c^	*P*_interaction_	Cases n (%)	HR (95% CI)^d^	*P*_trend_ ^c^	*P*_interaction_
Sex					.71				.02				.60
Men	1 703 928	563 (57.2)	1.01 (0.98‐1.04)	.47		145 (25.8)	0.92 (0.86‐0.99)	.04		418 (74.2)	1.03 (1.00‐1.06)	.04	
Women	3 751 579	421 (42.8)	1.03 (0.93‐1.14)	.56		79 (18.8)	1.07 (0.90‐1.27)	.45		342 (81.2)	1.02 (0.91‐1.15)	.74	
Smoking status					.17				.37				.30
Never	2 916 755	382 (38.8)	1.05 (1.00‐1.11)	.07		59 (15.5)	1.00 (0.86‐1.16)	.99		323 (84.5)	1.06 (1.00‐1.12)	.04	
Ever	2 516 949	602 (61.2)	1.01 (0.98‐1.04)	.65		165 (27.4)	0.93 (0.86‐1.00)	.06		437 (72.6)	1.03 (1.00‐1.06)	.09	
BMI					.37				.07				.89
<25 kg/m^2^	2 647 303	319 (32.4)	1.03 (0.99‐1.08)	.19		72 (22.6)	1.01 (0.92‐1.12)	.78		247 (77.4)	1.03 (0.98‐1.09)	.20	
≥25 kg/m^2^	2 808 204	665 (67.6)	1.01 (0.98‐1.04)	.68		152 (22.9)	0.90 (0.83‐0.98)	.02		513 (77.1)	1.03 (1.00‐1.06)	.06	

Abbreviations: CI, confidence interval; HR, hazard ratio.

^a^ Cardia (C16.0) and noncardia (C16.1‐6, C16.8, C16.9).

^b^ Adjusted for age, sex, education (primary school, technical school, secondary school, university), cigarette smoking (never, former >10 years since quitting, former ≤10 years since quitting; current <20 cigarettes/d, current ≥20 cigarettes/d, other), body mass index (kg/m^2^), total red and processed meat intake (g/d), fruit intake (g/d) and total energy from food not including alcoholic beverages (Kcal/d), and stratified by birth cohort (year of birth <1925, 5‐year categories for 1925 to 1964, ≥1965) and center (center in EPIC, two categories for individuals born in Australia/New Zealand/United Kingdom or Italy/Greece in MCCS).

^c^ Wald test from Cox regression models assessing linear trends for a 10 g/d increment in alcohol intake.

^d^ Adjusted for age, sex, education (primary school, technical school, secondary school, university), cigarette smoking (never, former >10 years since quitting, former ≤10 years since quitting; current <20 cigarettes/d, current ≥20 cigarettes/d, other), body mass index (kg/m^2^), total red and processed meat intake (g/d), fruit intake (g/d) and total energy from food not including alcoholic beverages (Kcal/d), and stratified by birth cohort (year of birth <1925, 5‐year categories for 1925 to 1964, ≥1965) and center (center in EPIC, two categories for individuals born in Australia/New Zealand/United Kingdom or Italy/Greece in MCCS); interaction terms were also fit for sex, cigarette smoking and education in the models.

HRs for lifetime alcohol intake did not differ appreciably in separate analyses of stomach cancers of specified noncardia sites (C16.1‐C16.6) and overlapping/unspecified stomach cancers (C16.8 and C16.9) (Supplementary Table 4). Differences in HRs between diffuse‐type and intestinal‐type stomach cancer were minimal (*P*_homogeneity_ = .97) (Supplementary Table 5). Baseline alcohol intake was not associated with incidence of stomach cancer or its subtypes (Table 2).

### 3.2 Alcohol intake trajectories and stomach cancer incidence

**FIGURE 2:**
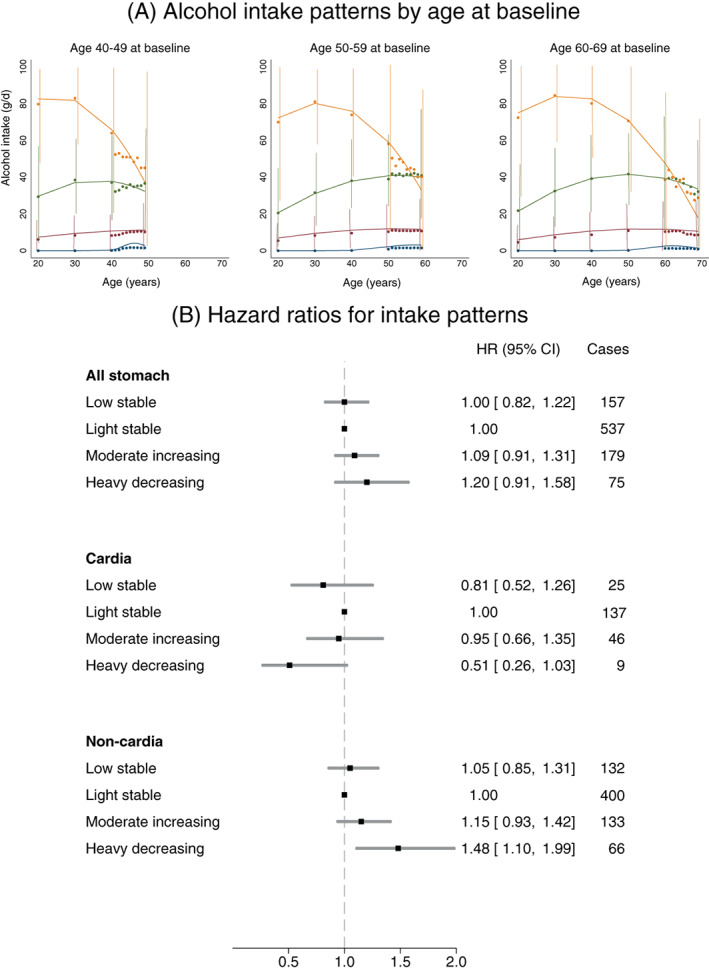
A, Patterns of alcohol intake during lifetime according to age at baseline (low stable, blue; light stable, red; moderate increasing, green; heavy decreasing, yellow) (circles represent average alcohol intake and vertical bars represent variation of alcohol intake between 10th and 90th percentile values at different assessment ages) and B, forest plot of adjusted hazard ratios (HRs) and 95% confidence intervals (CIs) for overall and site‐specific stomach cancer incidence according to alcohol intake pattern for all participants

Four patterns of alcohol intake over time, depicting *low stable*, *light stable*, *moderate increasing* and *heavy decreasing* intakes, were identified (Figure 2A). *Heavy decreasing* intake, compared to *light stable* intake, was positively associated with noncardia cancer (HR = 1.48, 95% CI: 1.10‐1.99); the corresponding HR for cancer of the gastric cardia was 0.51 (95% CI: 0.26‐1.03) (*P*_homogeneity_ = .02) (Figure 2B; Supplementary Table 6). This finding was consistent after excluding former drinkers at baseline (Supplementary Table 6).

### 3.3 Effect modification

There was no evidence that associations with lifetime alcohol intake differed between never and ever smokers for overall stomach cancer or by subsite and weak suggestive evidence that the inverse association for lifetime alcohol intake with cardia cancer was limited to overweight or obese individuals (*P*_interaction_ = .07) (Table 3). There was no evidence for an interaction between lifetime alcohol intake and *H pylori* infection status (*P*_interacton_ = .57; Table 4). The odds ratio for a 10 g/d increment in lifetime alcohol intake with adjustment for *H pylori* infection status was similar to the HR for noncardia cancer in the main analysis without adjustment for *H pylori* infection status.

**TABLE 4:** Odds ratios for noncardia stomach cancer by *Helicobacter pylori* status for a 10 g/d increment in lifetime alcohol intake for pooled nested case‐control study participants in the European Prospective Investigation into Cancer and Nutrition (EPIC) and the Melbourne Collaborative Cohort Study (MCCS)

	Noncardia stomach cancer cases (%)	Controls (%)	Median (IQR), g/d	OR (95% CI)^a^	*P*_interaction_
All participants	374 (100)	1163 (100)	9.4 (2.1‐24.5)	1.03 (0.97‐1.10)	
All participants^b^	374 (100)	1163 (100)	9.4 (2.1‐24.5)	1.03 (0.97‐1.10)	
By *Helicobacter pylori* status					.57
*Helicobacter pylori* positive	334 (89.3)	767 (66.0)	9.4 (1.5‐26.5)	1.04 (0.98‐1.10)	
*Helicobacter pylori* negative	40 (10.7)	396 (34.0)	9.3 (3.2‐19.1)	0.98 (0.82‐1.19)	

*Note*: Cases and controls matched on age group, sex, center and date of blood collection in EPIC and on year of birth, sex and country of birth in MCCS.

Abbreviations: CI, confidence interval; IQR, interquartile range; OR, odds ratios.

^a^ Adjusted for, education (primary school, technical school, secondary school, university), cigarette smoking (never, former, current), body mass index (kg/m^2^), total meat intake (g/d), fruit intake (g/d) and total energy from food (Kcal/d).

^b^ Additionally adjusted for *Helicobacter pylori* infection status.

### 3.4 Beverage‐specific intakes and stomach cancer incidence

There was no evidence of associations between individual beverage types and overall stomach cancer incidence (Supplementary Table 7). The HRs for a 10 g/d increment in lifetime wine intake for noncardia and cardia cancers were 1.03 (95% CI: 0.98‐1.08) and 0.91 (95% CI: 0.81‐1.01), respectively (*P*_homogeneity_ = .02) (Supplementary Table 8); HRs for noncardia cancer were 1.21 (95% CI: 0.83‐1.77), 1.36 (95% CI: 1.01‐1.84) and 1.56 (95% CI: 1.11‐2.19) for the highest category for beer (≥30 g/d), wine (≥30 g/d) and spirit (≥15 g/d) respectively, compared to 0.1 to 4.9 g/d.

### 3.5 Sensitivity analyses

The following HRs were observed when estimated study‐specific HRs for a 10 g/d increment in lifetime alcohol intake were pooled using random effects models, for overall stomach, cardia and noncardia cancer, respectively: 1.01 (95% CI: 0.99‐1.04), 0.93 (95% CI: 0.86‐1.00) and 1.03 (95% CI: 1.00‐1.06), thus displaying very similar estimates (and corresponding CIs) to aggregate‐level data. Associations between alcohol intake and overall and subtypes of stomach cancer incidence did not change appreciably when the first 2 years of follow‐up were excluded (Supplementary Tables 9 and 10) or when models were not adjusted for body mass index and dietary covariates.

## 4 DISCUSSION

In our study, we observed a weak positive dose‐dependent association for lifetime alcohol intake limited to noncardia stomach cancer, which did not differ appreciably between men and women or by levels of smoking, body mass index or *H pylori* infection status. We identified four alcohol intake patterns during the life course, with heavy drinking during early adulthood being associated with higher risk of noncardia cancer, compared to consistent light drinking. A weak inverse association for cancer of the gastric cardia with lifetime alcohol intake was also observed in men.

Study strengths include the prospective design, comprehensive assessment of alcohol intake over the life course based on intakes at different ages, and over 15 years of follow‐up on average. Additionally, the large study size with nearly half‐a‐million study participants and over one thousand incident stomach cancers enabled the examination of associations for subtypes and by sex, cigarette smoking and body fatness and we were also able to study individual alcoholic beverage types as well as heavy consumption. Information on *H pylori* status at baseline was available for subsamples of the studies. Among the several limitations is the use of self‐reported alcohol intake and exposure misclassification, hence bias in HR estimates cannot be ruled out. We also cannot completely rule out misclassification of stomach cancer subtypes, as 38.1% and 19.4% of tumors were not classified for site and histology, respectively. For instance in European settings and in Australia, nearly 50% of stomach cancers are coded as overlapping (C16.8) or unspecified (C16.9) for site,^34^ due to the difficulty in determining the origin of large tumors or those in the poorly defined distal boundary of the gastric cardia.^35^ We pooled overlapping or unspecified tumors with noncardia cancer consistent with other large cohort studies,^31^, ^32^ raising the possibility of misclassification, but sensitivity analyses showed that the HRs were not affected. In any case, contamination of overlapping and unspecified tumors by cardia cancer will move the HR for the pooled noncardia cancer toward the null that is, the HR will be underestimated.

Alcohol intake is classified as a probable cause of stomach cancer based on evidence for an association for intakes of 45 g/d or more, compared to abstention, mostly using data on alcohol intake at recruitment from case‐control studies.^5^ No association has been observed for women.^5^, ^36^ An earlier analysis of the EPIC study found increased risk of stomach cancer associated with heavy baseline alcohol intake^37^; other prospective studies did not report a similar finding.^31^, ^32^, ^38^ Similarly, evidence for an association between baseline alcohol intake and noncardia stomach cancer using prospective data remains inconclusive.^5^, ^37^, ^38^ The Shanghai Cohort Study previously observed an increased risk of stomach cancer associated with long‐term drinking in men, that is, a HR of 1.49 (95% CI: 1.01‐2.19) for those who drank four drinks per day or more for more than 30 years compared to nondrinkers,^39^ and also reported a HR of 1.51 (95% CI: 0.99‐2.32) for noncardia cancer for heavy drinkers compared to nondrinkers,^39^ similar to the present study. The association between lifetime alcohol intake and noncardia cancer risk was independent of *H pylori* infection status in contrast with previous findings^40^ or smoking status as in published data.^5^, ^41^

No previous observational study examined drinking trajectories in relation to stomach cancer. The association of noncardia cancer with heavy drinking during early adulthood using trajectories and the association with lifetime alcohol intake in our study are suggestive of early initiation and chronic progression of carcinogenesis linked to alcohol and its metabolites.^42^ Acetaldehyde, the toxic metabolite of ethanol in alcoholic beverages, is a carcinogen.^43^ Although the liver plays the major role in alcohol metabolism, first pass metabolism in the stomach also produces acetaldehyde,^44^ predominantly in the gastric mucosa in the body of stomach^45^ and more so in males, following high alcohol concentrations and when the stomach is full.^46^ The precise mechanisms leading to alcohol‐associated noncardia carcinogenesis, nonetheless, are far from being established. Associations of alcohol with stomach cancer risk may be mediated by retinoid metabolism, leading to adverse effects on cellular differentiation and apoptosis, the production of lipid peroxidation and oxygen free radicals, or by direct cellular injury and gene mutation by enhancing penetration of carcinogens into cells.^5^

Cancers of the gastric cardia and noncardia differ substantially in their patterns of incidence and etiology.^47^ While noncardia cancer is more common globally, cardia cancer is becoming increasingly common in high income countries where central obesity is also increasingly prevalent.^48^ The suggestive inverse association for cardia cancer with lifetime alcohol intake that was limited to men and to overweight or obese individuals in the present study cannot be explained mechanistically and could be due to chance. While studies have shown an inverse association for alcohol use with esophageal adenocarcinoma,^49^ a tumor similar to cardia cancer in terms of etiology and response to treatment, the Continuous Update Project of the World Cancer Research Fund/American Institute for Cancer Research found no evidence of an association.^5^ A previous meta‐analysis comparing drinkers with nondrinkers reported relative risks of 0.87 (95% CI: 0.74‐1.01) and 0.89 (95% CI: 0.76‐1.03) for adenocarcinoma of the esophagus and cardia cancer, respectively.^50^ Cardia cancers may be more impacted by mis‐classification than noncardia due to the difficulty in distinguishing tumors that are in close proximity to each other and tumors that often overgrow the gastroesophageal junction.^35^ In an EPIC subsample, 10% of cardia cancers were found to be esophageal adenocarcinomas after histopathologic review.^20^

In conclusion, lifetime alcohol intake was associated with increased risk of noncardia stomach cancer, independent of smoking, body mass index and *H pylori* infection status. Limiting alcohol use during lifetime, particularly avoiding heavy use during early adulthood, might help prevent noncardia stomach cancer.

## CONFLICT OF INTEREST

All authors have completed the ICMJE uniform disclosure form at www.icmje.org/coi_disclosure.pdf and declare: no support from any organization for the submitted work; no financial relationships with any organizations that might have an interest in the submitted work in the previous 3 years; and no other relationships or activities that could appear to have influenced the submitted work.

## IARC DISCLAIMER

Where authors are identified as personnel of the International Agency for Research on Cancer/World Health Organization, the authors alone are responsible for the views expressed in this article and they do not necessarily represent the decisions, policy or views of the International Agency for Research on Cancer/World Health Organization.

## ETHICS STATEMENT

The ethical review boards of the International Agency for Research on Cancer and all local institutions where participants had been recruited gave approval for the study. All participants gave written informed consent. The MCCS study protocol was approved by the Cancer Council Victoria Human Research Ethics Committee. Participants gave written informed consent to participate and for investigators to obtain access to their medical records.

## Supporting information


**Appendix S1**: Supporting Information

## Data Availability

Statistical code is available from the lead and the corresponding Authors. Access to EPIC data and biospecimens can be found at http://epic.iarc.fr/access/index.php. The MCCS data can be made available on request to pedigree@cancervic.org.au.
