# Supplementary material for: Lifetime alcohol intake, drinking patterns over time and risk of stomach cancer: A pooled analysis of data from two prospective cohort studies
Source: Int J Cancer. 2021 Feb 22;148(11):2759–73. doi: 10.1002/ijc.33504 (PMC9290950; doi:10.1002/ijc.33504)
Supplement: Supplementary file 1 — Appendix S1: Supporting Information [file IJC-148-2759-s001.pdf]

Lifetime alcohol intake, drinking patterns over time, and risk of stomach cancer: a pooled analysis of data from two prospective cohort studies

Harindra Jayasekara, Robert J. MacInnis, Leila Lujan Barroso, Ana-Lucia Mayen-Chacon, Amanda J Cross, Bengt Wallner, Domenico Palli, Fulvio Ricceri, Valeria Pala, Salvatore Panico, Rosario Tumino, Tilman Kühn, Rudolf Kaaks, Kostas Tsilidis, Maria-Jose Sánchez, Pilar Amiano, Eva Ardanaz, María Dolores Chirlaque López, Susana Merino, Joseph A. Rothwell, Marie-Christine Boutron-Ruault, Gianluca Severi, Hanna Sternby, Emily Sonestedt, Bas Bueno-de-Mesquita, Heiner Boeing, Ruth Travis, Torkjel M. Sandanger, Antonia Trichopoulou, Anna Karakatsani, Eleni Peppa, Anne Tjønneland, Yi Yang, Allison M. Hodge, Hazel Mitchell, Andrew Haydon, Robin Room, John L. Hopper, Elisabete Weiderpass, Marc J. Gunter, Elio Riboli, Graham G. Giles, Roger L. Milne, Antonio Agudo, Dallas R. English and Pietro Ferrari

Supplementary Table 1. Country-specific incidence rates of stomach cancer in the European Prospective Investigation into Cancer and Nutrition (EPIC) and the Melbourne Collaborative Cohort Study (MCCS).

Supplementary Table 2. Pooled and study-specific incidence rate of stomach cancer, person-years, the percentage of lifetime abstainers, and means (10th–90th percentile) of lifetime alcohol, beer, wine and spirits intakes (g/day) for participants in the European Prospective Investigation into Cancer and Nutrition (EPIC) and the Melbourne Collaborative Cohort Study (MCCS).

Supplementary Table 3. Hazard ratios for overall and site-specific stomach cancer for lifetime alcohol intake categories by sex in the European Prospective Investigation into Cancer and Nutrition (EPIC) and the Melbourne Collaborative Cohort Study (MCCS).

Supplementary Table 4. Hazard ratios for site-specific stomach cancer for baseline and lifetime alcohol intake in the European Prospective Investigation into Cancer and Nutrition (EPIC) and the Melbourne Collaborative Cohort Study (MCCS).

Supplementary Table 5. Hazard ratios for histologic subtypes of stomach cancer for baseline and lifetime alcohol intake in the European Prospective Investigation into Cancer and Nutrition (EPIC) and the Melbourne Collaborative Cohort Study (MCCS).

Supplementary Table 6. Hazard ratios for overall and site-specific stomach cancer by alcohol intake pattern during lifetime in the European Prospective Investigation into Cancer and Nutrition (EPIC) and the Melbourne Collaborative Cohort Study (MCCS).

Supplementary Table 7. Hazard ratios for overall stomach cancer for baseline and lifetime beverage-specific intake in the European Prospective Investigation into Cancer and Nutrition (EPIC) and the Melbourne Collaborative Cohort Study (MCCS).

Supplementary Table 8. Hazard ratios for site-specific stomach cancer for baseline and lifetime beverage-specific intake in the European Prospective Investigation into Cancer and Nutrition (EPIC) and the Melbourne Collaborative Cohort Study (MCCS).

Supplementary Table 9. Hazard ratios for overall stomach cancer for lifetime alcohol intake in the European Prospective Investigation into Cancer and Nutrition (EPIC) and the Melbourne Collaborative Cohort Study (MCCS) excluding first 2 years of follow-up.

Supplementary Table 10. Hazard ratios for anatomical and histologic subtypes of stomach cancer for lifetime alcohol intake in the European Prospective Investigation into Cancer and Nutrition (EPIC) and the Melbourne Collaborative Cohort Study (MCCS) excluding first 2 years of follow-up.

Supplementary Figure 1. Directed acyclic graph for association between alcohol intake and risk of stomach cancer

Supplementary Figure 2. Dose-response relationships for lifetime alcohol intake and risk of cancer of the gastric cardia and non-cardia stomach cancer. Hazard ratios, solid line; 95% confidence limits, dashed lines; reference line, grey dashed line.

Supplementary Table 1. Country-specific incidence rates of stomach cancer in the European Prospective Investigation into Cancer and Nutrition (EPIC) and the Melbourne Collaborative Cohort Study (MCCS).

	**Follow-up (person-years)**	**Stomach cancers (n, %)**	**Incidence rate (95% CI) (per 100,000 person-years)**
**EPIC**			
Country			
France[1]	811,486	13 (1.3)	1.6 (0.9 to 2.8)
Italy	626,651	144 (14.3)	23.0 (19.5 to 27.1)
Spain	633,133	141 (14.1)	22.3 (18.9 to 26.3)
United Kingdom	927,425	82 (8.2)	8.8 (7.1 to 11.0)
The Netherlands	521,105	70 (7.0)	13.4 (10.6 to 17.0)
Greece	266,994	67 (6.7)	25.1 (19.8 to 31.9)
Germany	503,091	124 (12.4)	24.6 (20.7 to 29.4)
Sweden	792,472	155 (15.4)	19.6 (16.7 to 22.9)
Denmark	811,848	172 (17.1)	21.2 (18.2 to 24.6)
Norway[1]	426,603	35 (3.5)	8.2 (5.9 to 11.4)
**MCCS**			
Country of birth			
Australia/New Zealand/United Kingdom	579,804	114 (51.4)	19.7 (16.4 to 23.6)
Italy/Greece	194,025	108 (48.6)	55.7 (46.1 to 67.2)

CI, confidence interval.

[1]Only women are included in France and Norway while some morphology codes are also not included in France.

Supplementary Table 2. Pooled and study-specific incidence rate of stomach cancer, person-years, the percentage of lifetime abstainers, and means (10th–90th percentile) of lifetime alcohol, beer, wine and spirits intakes (g/day) for participants in the European Prospective Investigation into Cancer and Nutrition (EPIC) and the Melbourne Collaborative Cohort Study (MCCS).

	**Follow-up (person-years)**	**Stomach cancers**				**Exposure to alcohol during lifetime**				
		**Cardia (C16.0) (n, %)**	**Non-cardia (C16.1–6, C16.8, C16.9) (n, %)**	**All (C16) (n, %)**	**All (C16) Incidence rate (95% CI)[1]**	**Abstainers (n, %)**	**Alcohol (g/day)[2]**	**Beer (g/day)[2]**	**Wine (g/day)[2]**	**Spirits (g/day)[2]**
EPIC										
Men (n=137,138)	1,910,647	153 (67.4)	403 (51.9)	556 (55.4)	29.1 (26.8 to 31.6)	1,692 (1.23)	18.4 (8.9 to 34.1)	4.9 (1.5 to 11.6)	5.5 (1.8 to 15.4)	2.4 (0.7 to 6.2)
Women (n=315,820)	4,410,161	74 (32.6)	373 (48.1)	447 (44.6)	10.1 (9.2 to 11.1)	25,678 (8.1)	5.0 (1.7 to 10.7)	0.7 (0.2 to 2.0)	3.00 (1.0 to 7.2)	0.6 (0.1 to 1.8)
All (n=452,958)	6,320,808	227 (100)	776 (100)	1,003 (100)	15.9 (14.9 to 16.9)	27,370 (6.0)	7.7 (2.7 to 17.2)	1.4 (0.3 to 5.0)	3.6 (1.2 to 8.9)	1.1 (0.2 to 3.3)
MCCS										
Men (n=15,804)	297,701	28 (59.6)	106 (60.6)	134 (60.4)	45.0 (38.0 to 53.3)	2,233 (14.1)	18.0 (8.0 to 33.3)	7.6 (2.7 to 18.7)	6.8 (2.2 to 15.0)	1.1 (0.3 to 3.1)
Women (n=22,952)	476,128	19 (40.4)	69 (39.4)	88 (39.6)	18.5 (15.0 to 22.8)	8,849 (38.5)	6.3 (2.1 to 13.5)	0.9 (0.2 to 2.9)	4.6 (1.4 to 11.0)	0.7 (0.2 to 2.1)
All (n=38,756)	773,829	47 (100)	175 (100)	222 (100)	28.7 (25.2 to 32.7)	11,082 (28.6)	10.7 (4.0 to 22.6)	4.0 (0.9 to 12.8)	5.6 (1.8 to 12.9)	0.9 (0.3 to 2.6)
Pooled										
Men (n=152,942)	2,208,348	181 (66.1)	509 (53.5)	690 (56.3)	31.2 (29.0 to 33.7)	3,925 (2.6)	18.4 (8.8 to 34.0)	5.2 (1.7 to 12.4)	5.6 (1.8 to 15.3)	2.3 (0.7 to 6.0)
Women (n=338,772)	4,886,289	93 (33.9)	442 (46.5)	535 (43.7)	10.9 (10.1 to 11.9)	34,527 (10.2)	5.1 (1.8 to 10.8)	0.7 (0.2 to 2.0)	3.1 (1.0 to 7.4)	0.6 (0.1 to 1.8)
All (n=491,714)	7,094,637	274 (100)	951 (100)	1,225 (100)	17.3 (16.3 to 18.3)	38,452 (7.8)	7.9 (2.8 to 17.6)	1.5 (0.4 to 5.5)	3.7 (1.2 to 9.1)	1.0 (0.2 to 3.3)

CI, confidence interval.

[1]per 100,000 person-years

[2]Values are medians and interquartile ranges for drinkers only.

Supplementary Table 3. Hazard ratios for overall and site-specific stomach cancer for lifetime alcohol intake categories by sex in the European Prospective Investigation into Cancer and Nutrition (EPIC) and the Melbourne Collaborative Cohort Study (MCCS).

	All stomach cancer		By subsite			
			Cancer of the gastric cardia*		Non-cardia cancer*	
	Cases n (%)	HR (95% CI)†	Cases n (%)	HR (95% CI)††	Cases n (%)	HR (95% CI)††
Men						
Abstainers	27 (4.8)	0.80 (0.51 to 1.26)	5 (3.5)	0.50 (0.26 to 0.98)	22 (5.2)	0.96 (0.59 to 1.55)
0.1-4.9	74 (13.1)	1.00	25 (17.2)	1.00	49 (11.7)	1.00
5-14.9	118 (21.0)	0.74 (0.55 to 0.99)	35 (24.1)	0.59 (0.37 to 0.94)	83 (19.9)	0.81 (0.58 to 1.15)
≥15	344 (61.1)	0.91 (0.71 to 1.18)	80 (55.2)	0.62 (0.39 to 0.96)	264 (63.2)	1.07 (0.79 to 1.46)
Women						
Abstainers	87 (20.6)	0.96 (0.73 to 1.26)	10 (12.7)	0.65 (0.35 to 1.20)	77 (22.5)	1.01 (0.76 to 1.35)
0.1-4.9	170 (40.4)	1.00	33 (41.8)	1.00	137 (40.0)	1.00
5-14.9	106 (25.2)	0.89 (0.70 to 1.14)	22 (27.8)	0.95 (0.60 to 1.51)	84 (24.6)	1.08 (0.76 to 1.53)
≥15	58 (13.8)	1.19 (0.88 to 1.61)	14 (17.7)	1.24 (0.67 to 2.29)	44 (12.9)	1.76 (1.00 to 3.09)

CI, confidence interval; HR, hazard ratio.
*Cardia (C16.0) and non-cardia (C16.1–6, C16.8, C16.9).
†Adjusted for age, sex, education (primary school, technical school, secondary school, university), cigarette smoking (never, former >10 years since quitting, former ≤10 years since quitting; current <20 cigarettes/day, current ≥20 cigarettes/day, other), body mass index (kg/m^2), total red and processed meat intake (g/day), fruit intake (g/day) and total energy from food not including alcoholic beverages (Kcal/day), and stratified by birth cohort (year of birth <1925, 5-year categories for 1925 to 1964, ≥1965) and center (center in EPIC, 2 categories for individuals born in Australia/New Zealand/United Kingdom or Italy/Greece in MCCS).
††Adjusted for age, sex, education (primary school, technical school, secondary school, university), cigarette smoking (never, former >10 years since quitting, former ≤10 years since quitting; current <20 cigarettes/day, current ≥20 cigarettes/day, other), body mass index (kg/m^2), total red and processed meat intake (g/day), fruit intake (g/day) and total energy from food not including alcoholic beverages (Kcal/day), and stratified by birth cohort (year of birth <1925, 5-year categories for 1925 to 1964, ≥1965) and center (center in EPIC, 2 categories for individuals born in Australia/New Zealand/United Kingdom or Italy/Greece in MCCS); interaction terms were also fit for sex, cigarette smoking and education in the models.

Supplementary Table 4. Hazard ratios for site-specific stomach cancer for baseline and lifetime alcohol intake in the European Prospective Investigation into Cancer and Nutrition (EPIC) and the Melbourne Collaborative Cohort Study (MCCS).

	**Baseline alcohol intake**				**Lifetime alcohol intake**			
	**Cases, n (%)**	**HR (95% CI)†**	p_{trend}[1]	$p_{homogeneity}$[2]	**Cases, n (%)**	**HR (95% CI)†**	p_{trend}[1]	$p_{homogeneity}$[2]
All								
For a 10 g/day increment				0.77				0.02
Cardia	274 (22.4)	1.01 (0.96 to 1.07)	0.63		224 (22.8)	0.93 (0.87 to 1.00)	0.06	
Non-cardia	484 (39.5)	0.99 (0.95 to 1.04)	0.80		391 (39.7)	1.02 (0.98 to 1.07)	0.26	
Overlapping/unspecified	467 (38.1)	1.02 (0.97 to 1.06)	0.48		369 (37.5)	1.04 (1.00 to 1.07)	0.06	
Intake categories				0.81				0.12
Cardia	274 (22.4)				224 (22.8)			
Abstainers	36 (13.1)	0.81 (0.54 to 1.23)			15 (6.7)	0.58 (0.33 to 1.04)		
0.1-4.9	69 (25.2)	1.00			58 (25.9)	1.00		
5-14.9	59 (21.5)	0.91 (0.64 to 1.29)			57 (25.5)	0.76 (0.52 to 1.10)		
15-29.9	50 (18.3)	1.03 (0.71 to 1.50)			58 (25.9)	0.98 (0.66 to 1.46)		
30-59.9	46 (16.8)	1.12 (0.75 to 1.65)			27 (12.0)	0.62 (0.38 to 1.02)		
≥60	14 (5.1)	0.91 (0.50 to 1.65)			9 (4.0)	0.48 (0.23 to 1.01)		
Non-cardia	484 (39.5)				391 (39.7)			
Abstainers	98 (20.3)	0.97 (0.74 to 1.27)			51 (13.0)	0.97 (0.68 to 1.38)		
0.1-4.9	134 (27.7)	1.00			97 (24.8)	1.00		
5-14.9	101 (20.9)	0.92 (0.71 to 1.20)			91 (23.3)	0.93 (0.70 to 1.25)		
15-29.9	78 (16.1)	1.06 (0.79 to 1.41)			80 (20.5)	1.22 (0.88 to 1.69)		
30-59.9	53 (10.9)	0.92 (0.66 to 1.29)			43 (11.0)	0.96 (0.64 to 1.44)		
≥60	20 (4.1)	0.95 (0.58 to 1.56)			29 (7.4)	1.46 (0.91 to 2.34)		
Overlapping/unspecified	467 (38.1)				369 (37.5)			
Abstainers	100 (21.4)	1.16 (0.88 to 1.53)			48 (13.0)	1.03 (0.72 to 1.48)		
0.1-4.9	118 (25.3)	1.00			89 (24.1)	1.00		
5-14.9	92 (19.7)	0.92 (0.70 to 1.21)			76 (20.6)	0.78 (0.57 to 1.07)		
15-29.9	66 (14.1)	0.93 (0.68 to 1.28)			69 (18.7)	0.99 (0.70 to 1.39)		
30-59.9	64 (13.7)	1.12 (0.81 to 1.55)			53 (14.4)	1.05 (0.72 to 1.55)		
≥60	27 (5.8)	1.28 (0.83 to 1.99)			34 (9.2)	1.52 (0.97 to 2.39)		
Men								
For a 10 g/day increment								
Cardia	181 (26.2)	1.01 (0.95 to 1.07)	0.80		145 (25.8)	0.93 (0.86 to 1.00)	0.04	
Non-cardia	244 (35.4)	0.99 (0.94 to 1.04)	0.61		203 (36.1)	1.02 (0.98 to 1.06)	0.33	

Overlapping/unspecified	265 (38.4)	1.01 (0.97 to 1.06)	0.60		215 (38.2)	1.04 (1.00 to 1.08)	0.04	
Women								
For a 10 g/day increment								
Cardia	93 (17.4)	1.06 (0.93 to 1.22)	0.36		79 (18.8)	1.05 (0.88 to 1.25)	0.61	
Non-cardia	240 (44.9)	1.03 (0.95 to 1.12)	0.52		188 (44.7)	1.06 (0.96 to 1.17)	0.27	
Overlapping/unspecified	202 (37.7)	1.05 (0.97 to 1.15)	0.23		154 (36.6)	1.08 (0.97 to 1.19)	0.17	

CI, confidence interval; HR, hazard ratio. Cardia (C16.0), non-cardia (C16.1–6) and overlapping/unspecified (C16.8–9).

†Adjusted for age, sex, education (primary school, technical school, secondary school, university), cigarette smoking (never, former >10 years since quitting, former ≤10 years since quitting; current <20 cigarettes/day, current ≥20 cigarettes/day, other), body mass index (kg/m^2), total red and processed meat intake (g/day), fruit intake (g/day) and total energy from food not including alcoholic beverages (Kcal/day), and stratified by birth cohort (year of birth <1925, 5-year categories for 1925 to 1964, ≥1965) and centre (centre in EPIC, 2 categories for individuals born in Australia/New Zealand/United Kingdom or Italy/Greece in MCCS); interaction terms were also fit for sex, cigarette smoking and education in the models.

[1]Wald test from Cox regression models assessing linear trends for a 10 g/day increment in alcohol intake.

[2]Test of homogeneity using the likelihood ratio test.

Supplementary Table 5. Hazard ratios for histologic subtypes of stomach cancer for baseline and lifetime alcohol intake in the European Prospective Investigation into Cancer and Nutrition (EPIC) and the Melbourne Collaborative Cohort Study (MCCS).

	**Baseline alcohol intake**				**Lifetime alcohol intake**			
	**Cases, n (%)**	**HR (95% CI)†**	p_{trend}[1]	$p_{homogeneity}$[2]	**Cases, n (%)**	**HR (95% CI)†**	p_{trend}[1]	$p_{homogeneity}$[2]
All								
For a 10 g/day increment				0.79				0.97
Diffuse-type	259 (21.1)	1.03 (0.97 to 1.09)	0.39		217 (22.1)	1.01 (0.96 to 1.07)	0.62	
Intestinal-type	729 (59.5)	1.00 (0.97 to 1.04)	0.86		556 (56.5)	1.01 (0.98 to 1.05)	0.56	
Intake categories				0.67				0.27
Diffuse-type	259 (21.1)				217 (22.1)			
Abstainers	45 (17.4)	0.90 (0.62 to 1.32)			22 (10.1)	0.66 (0.40 to1.07)		
0.1-4.9	68 (26.2)	1.00			65 (30.0)	1.00		
5-14.9	64 (24.7)	1.17 (0.83 to 1.66)			54 (24.9)	0.80 (0.56 to 1.17)		
15-29.9	36 (13.9)	1.01 (0.66 to 1.52)			39 (18.0)	0.86 (0.56 to 1.32)		
30-59.9	32 (12.4)	1.17 (0.75 to 1.81)			23 (10.6)	0.74 (0.44 to 1.26)		
≥60	14 (5.4)	1.47 (0.80 to 2.68)			14 (6.4)	1.03 (0.54 to 1.95)		
Intestinal-type	729 (59.5)				556 (56.5)			
Abstainers	140 (19.2)	1.07 (0.85 to 1.34)			63 (11.3)	1.04 (0.76 to 1.43)		
0.1-4.9	185 (25.4)	1.00			119 (21.4)	1.00		
5-14.9	148 (20.3)	0.90 (0.72 to 1.12)			136 (24.5)	0.98 (0.76 to 1.27)		
15-29.9	118 (16.2)	0.99 (0.78 to 1.26)			117 (21.0)	1.13 (0.85 to 1.49)		
30-59.9	101 (13.8)	1.02 (0.79 to 1.33)			75 (13.5)	0.98 (0.71 to 1.35)		
≥60	37 (5.1)	1.00 (0.69 to 1.44)			46 (8.3)	1.35 (0.92 to 1.97)		
Men				0.83				0.97
Diffuse-type	123 (17.8)	1.02 (0.95 to 1.09)	0.54		109 (19.4)	1.01 (0.95 to 1.07)	0.80	
Intestinal-type	448 (64.9)	1.00 (0.96 to 1.04)	0.98		348 (61.8)	1.01 (0.98 to 1.05)	0.57	
Women				0.92				0.46
Diffuse-type	136 (25.4)	1.06 (0.95 to 1.18)	0.33		108 (25.6)	1.07 (0.94 to 1.22)	0.31	
Intestinal-type	281 (52.5)	1.03 (0.95 to 1.12)	0.42		208 (49.4)	1.03 (0.93 to 1.14)	0.53	

CI, confidence interval; HR, hazard ratio.

† Adjusted for age, sex, education (primary school, technical school, secondary school, university), cigarette smoking (never, former >10 years since quitting, former ≤10 years since quitting; current <20 cigarettes/day, current ≥20 cigarettes/day, other), body mass index (kg/m^2), total red and processed meat intake (g/day), fruit intake (g/day) and total energy from food not including alcoholic beverages (Kcal/day), and stratified by birth cohort (year of birth <1925, 5-year categories for 1925 to 1964, ≥1965) and centre (centre in EPIC, 2 categories for individuals born in Australia/New Zealand/United Kingdom or Italy/Greece in MCCS); interaction terms were also fit for sex in the models.

[1]Wald test from Cox regression models assessing linear trends for a 10 g/day increment in alcohol intake.

[2]Test of homogeneity using the likelihood ratio test.

Diffuse-type (morphology codes 8145/3, 8490/3, 8142/3) and intestinal-type (8144/3, 8211/3, 8260/3, 8480/3, 8481/3, 8140/3) coded according to guidelines (Henson DE et al. Differential trends in the intestinal and diffuse types of gastric carcinoma in the United States, 1973-2000. *Arch Pathol Lab Med* 2004;128(7):765-70; Hu B et al. Gastric cancer: classification, histology and application of molecular pathology. *J Gastrointest Oncol* 2012;3(3):251-61); HRs for mixed/other/unknown-type (n=237; 19.4%) not reported.

Supplementary Table 6. Hazard ratios for overall and site-specific stomach cancer by alcohol intake pattern during lifetime in the European Prospective Investigation into Cancer and Nutrition (EPIC) and the Melbourne Collaborative Cohort Study (MCCS).

	All stomach cancer				By subsite				
					Cancer of the gastric cardia*		Non-cardia cancer*		$p_{homogeneity}$[2]
	**Person-years**	**Cases n (%)**	**HR (95% CI)†**	p_{trend}[1]	**Cases n (%)**	**HR (95% CI)†**	**Cases n (%)**	**HR (95% CI)†**	
All									
Alcohol intake patterns				0.18					0.02
Low stable	991,070	157 (16.6)	1.00 (0.82 to 1.22)		25 (11.5)	0.81 (0.52 to 1.26)	132 (18.1)	1.05 (0.85 to 1.31)	
Light stable	3,148,833	537 (56.6)	1.00		137 (63.1)	1.00	400 (54.7)	1.00	
Moderate increasing	562,367	179 (18.9)	1.09 (0.91 to 1.31)		46 (21.2)	0.95 (0.66 to 1.35)	133 (18.2)	1.15 (0.93 to 1.42)	
Heavy decreasing	149,037	75 (7.9)	1.20 (0.91 to 1.58)		9 (4.2)	0.51 (0.26 to 1.03)	66 (9.0)	1.48 (1.10 to 1.99)	
Excluding quitters at baseline									
Alcohol intake patterns				0.16					0.04
Low stable	982,451	155 (17.6)	1.00 (0.82 to 1.23)		24 (11.9)	0.78 (0.50 to 1.24)	131 (19.4)	1.06 (0.84 to 1.32)	
Light stable	2,899,676	484 (55.1)	1.00		125 (61.9)	1.00	359 (53.1)	1.00	
Moderate increasing	546,712	170 (19.4)	1.09 (0.90 to 1.32)		44 (21.8)	0.94 (0.66 to 1.36)	126 (18.6)	1.15 (0.92 to 1.43)	
Heavy decreasing	136,561	69 (7.9)	1.26 (0.94 to 1.68)		9 (4.4)	0.58 (0.29 to 1.17)	60 (8.9)	1.54 (1.12 to 2.10)	

CI, confidence interval; HR, hazard ratio.

*Cardia (C16.0) and non-cardia (C16.1–6, C16.8, C16.9).

†Adjusted for age, sex, education (primary school, technical school, secondary school, university), cigarette smoking (never, former >10 years since quitting, former ≤10 years since quitting; current <20 cigarettes/day, current ≥20 cigarettes/day, other), body mass index (kg/m^2), total red and processed meat intake (g/day), fruit intake (g/day) and total energy from food not including alcoholic beverages (Kcal/day), and stratified by birth cohort (year of birth <1925, 5-year categories for 1925 to 1964, ≥1965) and center (center in EPIC, 2 categories for individuals born in Australia/New Zealand/United Kingdom or Italy/Greece in MCCS).

[1]Wald test from Cox regression models assessing linear trends for intake categories as a continuous measure.

[2]Test of homogeneity using the likelihood ratio test.

Supplementary Table 7. Hazard ratios for overall stomach cancer for baseline and lifetime beverage-specific intake in the European Prospective Investigation into Cancer and Nutrition (EPIC) and the Melbourne Collaborative Cohort Study (MCCS).

	**Baseline intake**					**Lifetime intake**				
	**Person-years**	**Cases, n (%)**	**HR (95% CI)‡**	**HR (95% CI)†**	p_{trend}[1]	**Person-years**	**Cases, n (%)**	**HR (95% CI)‡**	**HR (95% CI)†**	p_{trend}[1]
**Beer**										
For a 10 g/day increment	7,094,044	1,225 (100)	1.06 (1.00 to 1.11)	1.03 (0.98 to 1.08)	0.31	5,455,484	984 (100)	1.05 (1.00 to 1.11)	1.01 (0.95 to 1.07)	0.70
Intake categories					0.77					0.81
Abstainers	2,898,852	499 (40.7)	1.03 (0.89 to 1.19)	1.02 (0.88 to 1.17)		1,850,723	291 (29.6)	0.91 (0.77 to 1.08)	0.91 (0.77 to 1.07)	
0.1-4.9	3,064,738	458 (37.4)	1.00	1.00		2,656,325	430 (43.7)	1.00	1.00	
5-14.9	788,668	175 (14.3)	1.04 (0.86 to 1.26)	1.02 (0.85 to 1.24)		638,557	164 (16.6)	1.10 (0.90 to 1.33)	1.04 (0.85 to 1.26)	
15-29.9	173,965	35 (2.9)	0.87 (0.61 to 1.24)	0.82 (0.57 to 1.16)		211,973	59 (6.0)	1.02 (0.76 to 1.36)	0.89 (0.67 to 1.19)	
≥30	167,821	58 (4.7)	1.35 (1.01 to 1.81)	1.15 (0.86 to 1.55)		97,906	40 (4.1)	1.32 (0.94 to 1.86)	1.07 (0.76 to 1.51)	
**Wine**										
For a 10 g/day increment	7,093,386	1,225 (100)	1.00 (0.96 to 1.05)	1.00 (0.96 to 1.04)	0.89	5,455,484	984 (100)	1.01 (0.97 to 1.06)	1.00 (0.96 to 1.05)	0.91
Intake categories					0.79					0.20
Abstainers	1,814,578	367 (30.0)	1.04 (0.89 to 1.21)	1.01 (0.86 to 1.17)		938,319	200 (20.3)	1.22 (1.01 to 1.47)	1.19 (0.98 to 1.43)	
0.1-4.9	2,697,959	394 (32.1)	1.00	1.00		2,563,270	374 (38.0)	1.00	1.00	
5-14.9	1,623,216	229 (18.7)	0.90 (0.76 to 1.06)	0.94 (0.79 to 1.12)		1,285,463	216 (22.0)	1.10 (0.92 to 1.31)	1.14 (0.96 to 1.35)	
15-29.9	621,219	138 (11.3)	1.11 (0.90 to 1.35)	1.15 (0.94 to 1.41)		449,243	106 (10.8)	1.12 (0.89 to 1.41)	1.13 (0.89 to 1.43)	
≥30	336,414	97 (7.9)	1.00 (0.79 to 1.28)	0.98 (0.76 to 1.25)		219,189	88 (8.9)	1.21 (0.92 to 1.59)	1.13 (0.86 to 1.49)	
**Spirit**										
For a 10 g/day increment	7,094,048	1,225 (100)	1.02 (0.91 to 1.14)	0.98 (0.87 to 1.10)	0.69	5,455,502	984 (100)	1.06 (1.02 to 1.11)	1.04 (0.99 to 1.09)	0.13
Intake categories					0.59					0.06
Abstainers	3,545,068	602 (49.1)	1.06 (0.92 to 1.22)	1.04 (0.91 to 1.20)		2,065,835	346 (35.1)	1.07 (0.91 to 1.26)	1.07 (0.91 to 1.26)	
0.1-4.9	3,133,153	505 (41.2)	1.00	1.00		2,814,599	475 (48.3)	1.00	1.00	
5-14.9	320,226	94 (7.7)	1.21 (0.96 to 1.52)	1.15 (0.92 to 1.45)		436,671	115 (11.7)	1.24 (1.00 to 1.53)	1.16 (0.94 to 1.44)	
≥15	95,601	24 (2.0)	0.98 (0.65 to 1.49)	0.87 (0.57 to 1.32)		138,397	48 (4.9)	1.48 (1.09 to 2.03)	1.26 (0.92 to 1.73)	

CI, confidence interval; HR, hazard ratio.

‡Adjusted for age and sex, and stratified by birth cohort (year of birth <1925, 5-year categories for 1925 to 1964, ≥1965) and centre (centre in EPIC, 2 categories for individuals born in Australia/New Zealand/United Kingdom or Italy/Greece in MCCS).

†Adjusted for age, sex, education (primary school, technical school, secondary school, university), cigarette smoking (never, former >10 years since quitting, former ≤10 years since quitting; current <20 cigarettes/day, current ≥20 cigarettes/day, other), body mass index (kg/m^2), total red and processed meat intake (g/day), fruit intake (g/day) and total energy from food not including alcoholic beverages (Kcal/day), and stratified by birth cohort (year of birth <1925, 5-year categories for 1925 to 1964, ≥1965) and centre (centre in EPIC, 2 categories for individuals born in Australia/New Zealand/United Kingdom or Italy/Greece in MCCS).

[1]Wald test from Cox regression models assessing linear trends for a 10 g/day increment in alcohol intake and for intake categories as a continuous measure.

Supplementary Table 8. Hazard ratios for site-specific stomach cancer for baseline and lifetime beverage-specific intake in the European Prospective Investigation into Cancer and Nutrition (EPIC) and the Melbourne Collaborative Cohort Study (MCCS).

	**Baseline intake**				**Lifetime intake**			
	**Cases, n (%)**	**HR (95% CI)†**	p_{trend}[1]	$p_{homogeneity}$[2]	**Cases, n (%)**	**HR (95% CI)†**	p_{trend}[1]	$p_{homogeneity}$[2]
**Beer**								
For a 10 g/day increment				0.22				0.25
Cardia	274 (22.4)	1.07 (0.99 to 1.16)	0.10		224 (22.8)	0.95 (0.83 to 1.08)	0.43	
Non-cardia	951 (77.6)	1.00 (0.94 to 1.07)	0.89		760 (77.2)	1.03 (0.96 to 1.10)	0.39	
**Wine**								
For a 10 g/day increment				0.63				0.02
Cardia	274 (22.4)	0.99 (0.91 to 1.07)	0.76		224 (22.8)	0.91 (0.81 to 1.01)	0.07	
Non-cardia	951 (77.6)	1.01 (0.96 to 1.06)	0.70		760 (77.2)	1.03 (0.98 to 1.08)	0.26	
**Spirit**								
For a 10 g/day increment				0.05				0.23
Cardia	274 (22.4)	0.76 (0.55 to 1.05)	0.09		224 (22.8)	0.96 (0.82 to 1.13)	0.64	
Non-cardia	951 (77.6)	1.03 (0.91 to 1.16)	0.61		760 (77.2)	1.05 (1.00 to 1.10)	0.04	

CI, confidence interval; HR, hazard ratio. Cardia (C16.0) and non-cardia (C16.1–6, C16.8, C16.9).
† Adjusted for age, sex, education (primary school, technical school, secondary school, university), cigarette smoking (never, former >10 years since quitting, former ≤10 years since quitting; current <20 cigarettes/day, current ≥20 cigarettes/day, other), body mass index (kg/m^2), total red and processed meat intake (g/day), fruit intake (g/day) and total energy from food not including alcoholic beverages (Kcal/day), and stratified by birth cohort (year of birth <1925, 5-year categories for 1925 to 1964, ≥1965) and centre (centre in EPIC, 2 categories for individuals born in Australia/New Zealand/United Kingdom or Italy/Greece in MCCS); interaction terms were also fit for sex, cigarette smoking and education in the models.
[1]Wald test from Cox regression models assessing linear trends for a 10 g/day increment in alcohol intake.
[2]Test of homogeneity using the likelihood ratio test.

Supplementary Table 9. Hazard ratios for overall stomach cancer for lifetime alcohol intake in the European Prospective Investigation into Cancer and Nutrition (EPIC) and the Melbourne Collaborative Cohort Study (MCCS) excluding first 2 years of follow-up.

	**Person-years**	**Cases, n (%)**	**HR (95% CI)†**	p_{trend}[1]
For a 10 g/day increment	4,696,005	905 (100)	1.02 (0.99 to 1.04)	0.28
Comparing drinkers with abstainers				
Abstainers	528,130	104 (11.5)	1.00	
Drinkers	4,167,875	801 (88.5)	1.08 (0.86 to 1.35)	0.53
Intake categories				0.77
Abstainers	528,130	104 (11.5)	0.88 (0.69 to 1.13)	
0.1-4.9	1,539,471	226 (25.0)	1.00	
5-14.9	1,376,339	202 (22.3)	0.81 (0.66 to 0.98)	
15-29.9	756,679	194 (21.5)	1.07 (0.87 to 1.32)	
30-59.9	372,525	109 (12.0)	0.84 (0.65 to 1.09)	
≥60	122,861	70 (7.7)	1.22 (0.90 to 1.67)	

CI, confidence interval; HR, hazard ratio.

†Adjusted for age, sex, education (primary school, technical school, secondary school, university), cigarette smoking (never, former >10 years since quitting, former ≤10 years since quitting; current <20 cigarettes/day, current ≥20 cigarettes/day, other), body mass index (kg/m^2), total red and processed meat intake (g/day), fruit intake (g/day) and total energy from food not including alcoholic beverages (Kcal/day), and stratified by birth cohort (year of birth <1925, 5-year categories for 1925 to 1964, ≥1965) and centre (centre in EPIC, 2 categories for individuals born in Australia/New Zealand/United Kingdom or Italy/Greece in MCCS).

[1]Wald test from Cox regression models assessing linear trends for a 10 g/day increment in alcohol intake and for intake categories as a continuous measure.

Supplementary Table 10. Hazard ratios for anatomical and histologic subtypes of stomach cancer for lifetime alcohol intake in the European Prospective Investigation into Cancer and Nutrition (EPIC) and the Melbourne Collaborative Cohort Study (MCCS) excluding first 2 years of follow-up.

	Cases, n (%)	HR (95% CI)†	p_{trend}[1]	$p_{homogeneity}$[2]
Anatomical site[3]				
For a 10 g/day increment				0.007
Cardia	208 (23.0)	0.94 (0.87 to 1.01)	0.08	
Non-cardia	697 (77.0)	1.03 (1.00 to 1.06)	0.02	
Intake categories				0.04
Cardia	208 (23.0)			
Abstainers	15 (7.2)	0.61 (0.34 to 1.09)		
0.1-4.9	54 (26.0)	1.00		
5-14.9	50 (24.0)	0.71 (0.48 to 1.06)		
15-29.9	57 (27.4)	1.04 (0.69 to 1.56)		
30-59.9	23 (11.1)	0.57 (0.34 to 0.97)		
≥60	9 (4.3)	0.52 (0.25 to 1.10)		
Non-cardia	697 (77.0)			
Abstainers	89 (12.8)	0.95 (0.73 to 1.25)		
0.1-4.9	172 (24.7)	1.00		
5-14.9	152 (21.8)	0.83 (0.67 to 1.05)		
15-29.9	137 (19.7)	1.07 (0.84 to 1.37)		
30-59.9	86 (12.3)	0.95 (0.71 to 1.28)		
≥60	61 (8.7)	1.52 (1.08 to 2.14)		
Histologic subtype[4]				
For a 10 g/day increment				0.99
Diffuse-type	191 (21.1)	1.01 (0.95 to 1.08)	0.68	
Intestinal-type	517 (57.1)	1.02 (0.98 to 1.05)	0.37	
Intake categories				0.36
Diffuse-type	191 (21.1)			
Abstainers	21 (11.0)	0.70 (0.42 to 1.17)		
0.1-4.9	57 (29.8)	1.00		
5-14.9	48 (25.1)	0.80 (0.54 to 1.19)		
15-29.9	33 (17.3)	0.80 (0.50 to 1.27)		
30-59.9	19 (10.0)	0.67 (0.38 to 1.18)		
≥60	13 (6.8)	1.04 (0.54 to 2.02)		
Intestinal-type	517 (57.1)			

Abstainers	56 (10.8)	0.95 (0.68 to 1.32)		
0.1-4.9	114 (22.1)	1.00		
5-14.9	122 (23.6)	0.91 (0.70 to 1.18)		
15-29.9	111 (21.5)	1.09 (0.82 to 1.45)		
30-59.9	68 (13.1)	0.91 (0.65 to 1.26)		
≥60	46 (8.9)	1.37 (0.93 to 2.02)		

CI, confidence interval; HR, hazard ratio.
† Adjusted for age, sex, education (primary school, technical school, secondary school, university), cigarette smoking (never, former >10 years since quitting, former ≤10 years since quitting; current <20 cigarettes/day, current ≥20 cigarettes/day, other), body mass index (kg/m^2), total red and processed meat intake (g/day), fruit intake (g/day) and total energy from food not including alcoholic beverages (Kcal/day), and stratified by birth cohort (year of birth <1925, 5-year categories for 1925 to 1964, ≥1965) and centre (centre in EPIC, 2 categories for individuals born in Australia/New Zealand/United Kingdom or Italy/Greece in MCCS); interaction terms were also fit for sex, cigarette smoking and education in the models.
[1]Wald test from Cox regression models assessing linear trends for a 10 g/day increment in alcohol intake.
[2]Test of homogeneity using the likelihood ratio test.
[3]Cardia (C16.0) and non-cardia (C16.1–6, C16.8, C16.9).
[4]Diffuse-type (morphology codes 8145/3, 8490/3, 8142/3) and intestinal-type (8144/3, 8211/3, 8260/3, 8480/3, 8481/3, 8140/3) coded according to guidelines (Henson DE et al. Differential trends in the intestinal and diffuse types of gastric carcinoma in the United States, 1973-2000. *Arch Pathol Lab Med* 2004;128(7):765-70; Hu B et al. Gastric cancer: classification, histology and application of molecular pathology. *J Gastrointest Oncol* 2012;3(3):251-61); HRs for mixed/other/unknown-type (n=222; 19.6%) not reported.

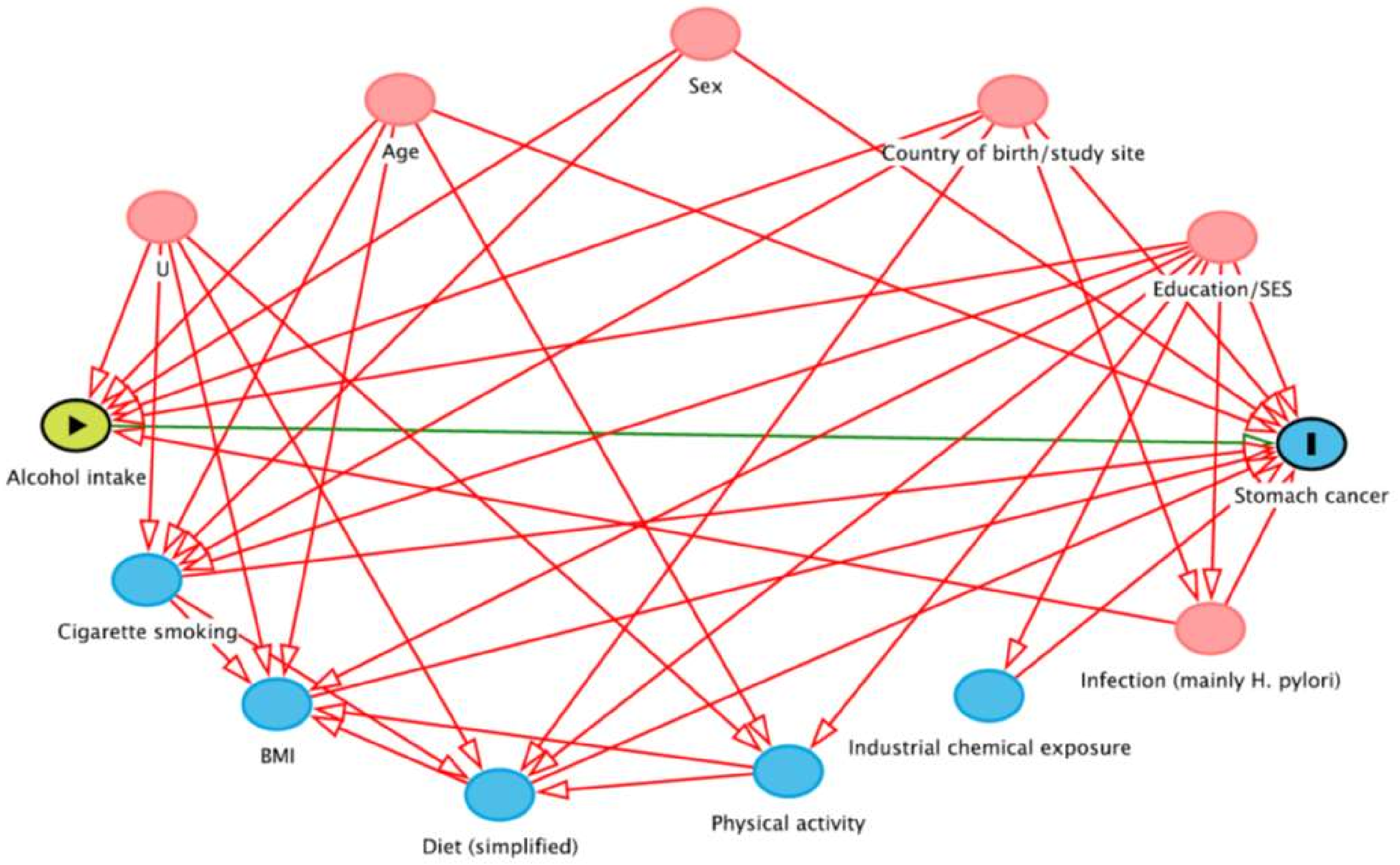

Supplementary Figure 1. Directed acyclic graph for association between alcohol intake and risk of stomach cancer
[Drawn using http://www.dagitty.net/ software: Textor J, van der Zander B, Gilthorpe MS, et al: Robust causal inference using directed acyclic graphs: the R package 'dagitty'. Int J Epidemiol 2016; 45(6): 1887–94]

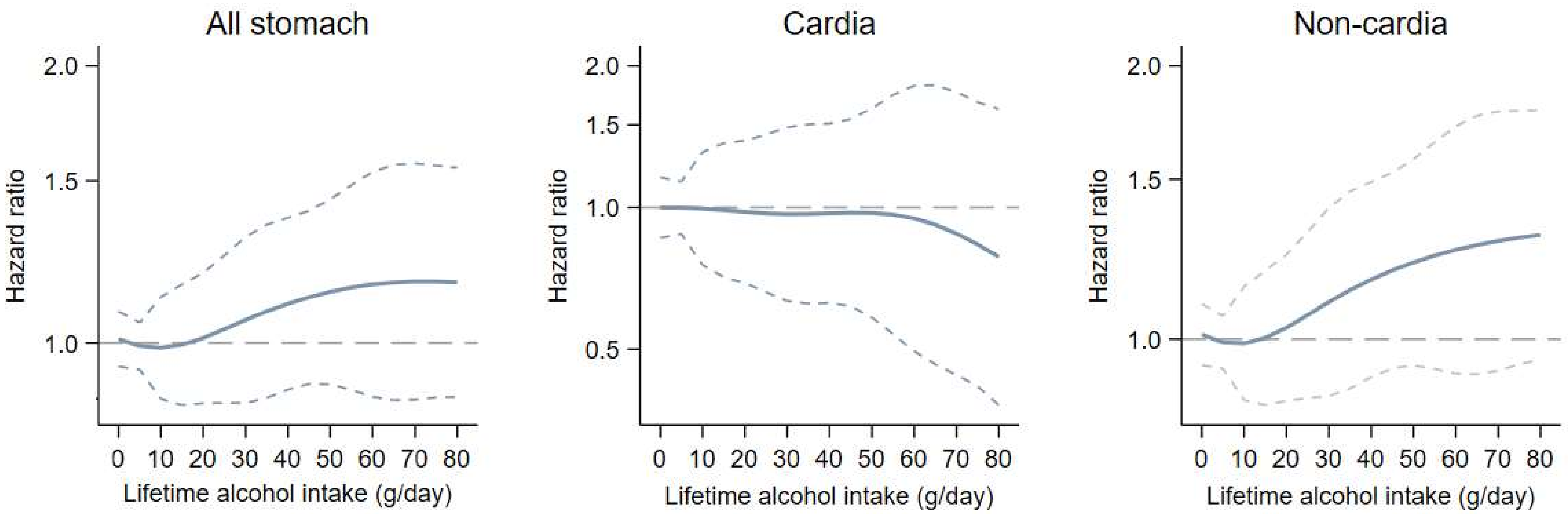

Supplementary Figure 2. Dose-response relationships for lifetime alcohol intake and risk of cancer of the gastric cardia and non-cardia stomach cancer. Hazard ratios, solid line; 95% confidence limits, dashed lines; reference line, grey dashed line.
